# A Meta-Analysis of Computerized Tomography-Based Radiomics for the Diagnosis of COVID-19 and Viral Pneumonia

**DOI:** 10.3390/diagnostics11060991

**Published:** 2021-05-29

**Authors:** Yung-Shuo Kao, Kun-Te Lin

**Affiliations:** 1 Department of Radiation Oncology, China Medical University Hospital, Taichung 404, Taiwan; codingforlifetime@gmail.com; 2 Department of Emergency Medicine, Changhua Christian Hospital, Changhua 500, Taiwan

**Keywords:** COVID-19, radiomics, meta-analysis

## Abstract

Introduction: Coronavirus disease 2019 (COVID-19) led to a global pandemic. Although reverse transcription polymerase chain reaction (RT-PCR) of viral nucleic acid is the gold standard for COVID-19 diagnosis, its sensitivity was found to not be high enough in many reports. As radiomics-based diagnosis research has recently emerged, we aimed to use computerized tomography (CT)-based radiomics models to differentiate COVID-19 pneumonia from other viral pneumonia infections. Materials and methods: This study was performed according to the preferred reporting items for systematic review and meta-analysis diagnostic test accuracy studies (PRISMA-DTA) guidelines. The Pubmed, Cochrane, and Embase databases were searched. The pooled sensitivity and pooled specificity were calculated. A summary receiver operating characteristic (sROC) curve was constructed. The study quality was evaluated based on the radiomics quality score. Results: A total of 10,300 patients were involved in this meta-analysis. The radiomics quality score ranged from 13 to 16 (maximum score: 36). The pooled sensitivity was 0.885 (95% CI: 0.818–0.929), and the pooled specificity was 0.811 (95% CI: 0.667–0.902). The pooled AUC was 906. Conclusion: Our meta-analysis showed that CT-based radiomics feature models can successfully differentiate COVID-19 from other viral pneumonias.

## 1. Introduction

Coronavirus disease 2019 (COVID-19) led to a global pandemic featuring a highly contagious disease which has led to millions of deaths worldwide. Although reverse transcription polymerase chain reaction (RT-PCR) of viral nucleic acid is the gold standard for COVID-19 diagnosis [1], its sensitivity was found to not be high enough in many reports [2,3,4].

On the other hand, chest x-ray (CXR) and computerized tomography (CT) are helpful in the diagnosis of COVID-19 pneumonia [5,6,7]. According to recent experience, lung image findings are observed earlier than clinical manifestations, highlighting the importance of image exams for screening pneumonias [8]. Early diagnosis can also reduce disease transmission risk and prevent the endemic of COVID-19 [9]. CXR is convenient, easily accessible, and can avoid cross contamination between users. Although the sensitivity of CXR is lower than CT in diagnosing COVID-19 pneumonia. With artificial intelligence machine learning model assistance, CXR can achieve an improved COVID-19 diagnostic rate [10,11,12].

However, the power of generalization is low when the machine learning model was trained on sets of heterogeneous images. The absence of an adequate evaluation protocol also limited the artificial intelligence assistance in CXR and its utility in clinical settings [13]. Nonetheless, CXR is useful to monitor disease progression in unstable patients.

In previous studies, computerized tomography (CT) had noninferior sensitivity to the RT-PCR test in diagnosing COVID-19 pneumonia [2,5]. However, CT images have an inevitable misdiagnosis rate of COVID-19 if they are evaluated by humans [14]. Radiomics, a non-invasive machine learning technology, as evaluated by computerized quantitative analysis, can extract the statistics, shape, or texture features of images. Previous studies showed that radiomics plays an important role in tumor diagnosis and treatment [15,16]. Recently, radiomics models on CT images were shown to be helpful in predicting COVID-19 prognosis, hospital stay, and disease severity [17,18,19].

Previous studies showed that artificial intelligence (AI) could distinguish COVID-19 pneumonia from pneumonias caused by other pathogens [3,20,21]. However, most published reports did not individually compare COVID-19 with other viral pneumonia infections. The differentiation of COVID-19 and other viral pneumonia infections is challenging in clinical practice. Pneumonia caused by viral infections presents similar clinical symptoms [22,23,24]. Furthermore, the CT image features are also similar in COVID-19 and other viral pneumonia infections [25]. In contrast, radiomics can transform conventional medical images into quantitative and high-dimensional data analysis [26]. CT radiomics can be useful to discriminate COVID-19 from other pulmonary ground-glass opacity lesions [27]. However, there are no meta-analyses comparing the differentiation of COVID-19 from other viral pneumonias using radiomics. Therefore, in this meta-analysis, we aimed to use CT-based radiomics models to differentiate COVID-19 pneumonia from other viral pneumonia infections.

## 2. Materials and Methods

### 2.1. Study Protocol and Literature Search

This study was performed according to the preferred reporting items for systematic review and meta-analysis diagnostic test accuracy studies (PRISMA-DTA) guidelines [28]. Three databases (Pubmed, Cochrane Library, and Embase) were independently searched by two authors for articles published before 26 February 2021 using the following keywords: (“COVID-19” OR “SARV-COV-2”) AND (“radiomics” OR “artificial intelligence”) AND “computerized tomography”.

The inclusion criteria were as follows:

Studies using radiomics methods to differentiate COVID-19 and other viral pneumonia infections according to CT scans:

1. Articles written in English;
2. Full text available.

The exclusion criteria were as follows:

1. Studies using only deep learning features;
2. Conference papers or studies with only the abstract available.

### 2.2. Workflow of the Radiomics Study

In the selected studies, the radiomics-based machine learning process was similar. First, the CT images acquisition and region-of-interest segmentation were performed by radiologists. Next, the radiomics feature extraction, model training and cross validation were performed by artificial intelligence. Finally, the data analysis and clinical application was evaluated by clinicians.

### 2.3. Data Extraction

The main outcome was set as the highest area under the curve (AUC) in the validation dataset. In the absence of an external validation set, we chose the value from the cross-validation process or hold-out dataset. We also extracted the true positive (TP), false positive (FP), true negative (TN), and false negative (FN) values from the literature. Furthermore, we gathered the name of the first author, the nation of the first author, the publication year of the study, the region of interest (ROI), the patient number, and other characteristics from the selected studies.

### 2.4. Statistical Analysis

Pooled analysis was carried out using a random effects model. The pooled sensitivity and pooled specificity were calculated. A forest plot and summary receiver operating characteristic (ROC) plot were created. The heterogeneity was assessed using the chi-square test. The statistical analysis was performed with the R language using R studio.

### 2.5. Bias Assessment

The publication bias was assessed using a funnel plot. Egger’s test was only conducted if the number of included articles was more than 10. The statistical analysis was performed with the R language using R studio.

### 2.6. Quality Assessment

The RQS (radiomics quality score) was used to evaluate the chosen studies [29]. Two authors independently scored the table. Any inconsistencies between the authors were resolved by consensus.

## 3. Results

### 3.1. Literature Collection

**Figure 1:**
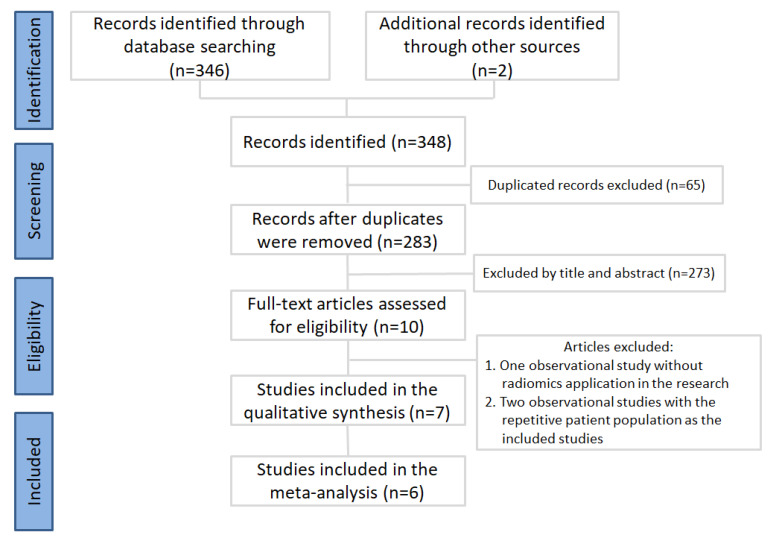
Inclusion process for the identified studies.

First, we collected a total of 348 articles. After duplicate removal, 283 publications were selected for meticulous evaluation. After assessment of the title and abstracts, ten publications were selected, and their full texts were retrieved. One observational study [30] without radiomics application in the research and two observational studies [31,32] with a repetitive patient population were excluded. After the article selection process, seven articles were used in the qualitative analysis [33,34,35,36,37,38,39], and six articles were further used in the meta-analysis. The PRISMA flowchart is provided in Figure 1. Details of the selected studies are listed in Table 1. Only the six studies were used in the meta-analysis [33,34,35,36,37,38]. Wang’s study [39] was excluded due to the predictions being made on the basis of the CT slice number. A total of 10,300 patients were involved in this meta-analysis. Among them, 3587 patients had COVID-19 pneumonia.

### 3.2. Quality Assessment and Workflow of the Radiomics Study

**Figure 2:**
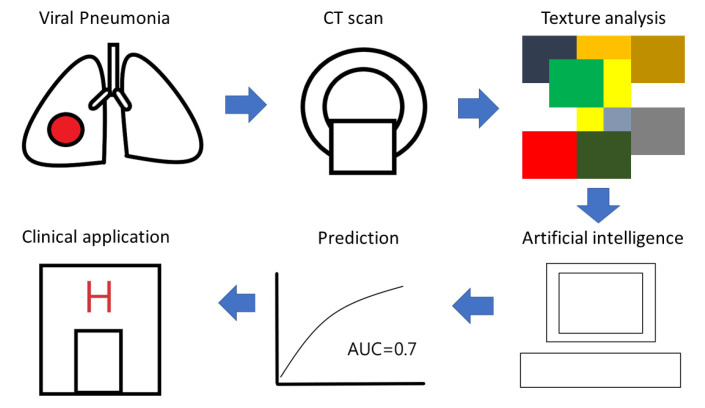
Workflow of the radiomics study.

The RQS table is provided in Table 2. The score range of included studies was 13 to 16 (maximum score: 36). None of the evaluated articles performed a phantom study, discussed biological correlates, conducted the study in a prospective design, or performed a cost-effectiveness analysis. The radiomics-based machine learning process workflow was shown in Figure 2.

### 3.3. Statistical Analysis

**Figure 3:**
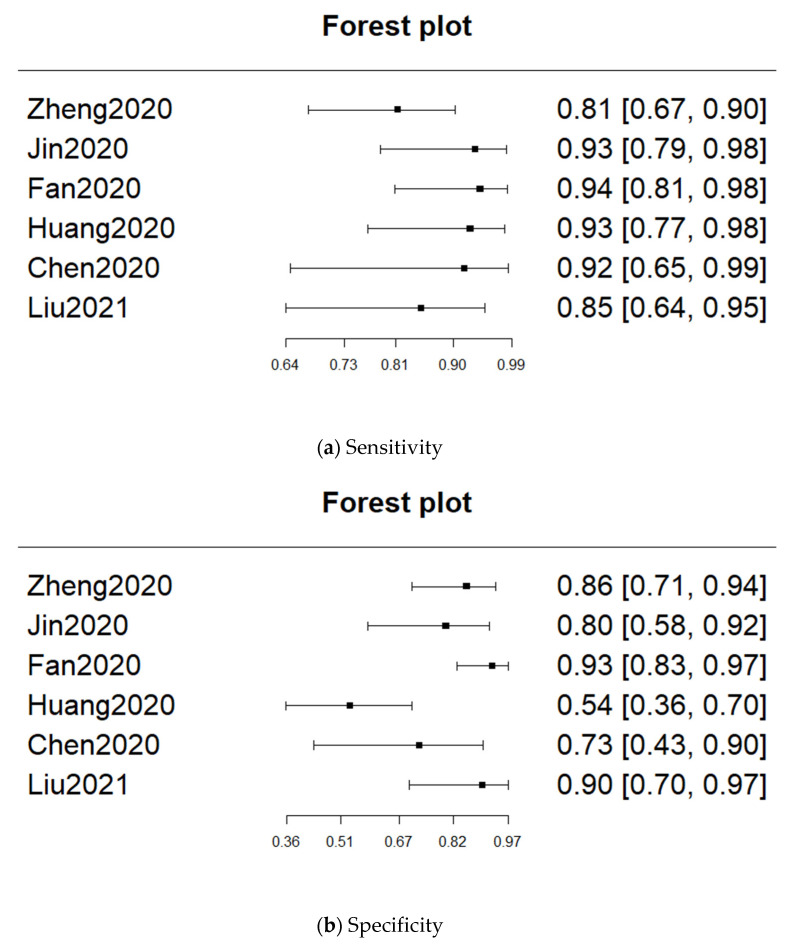
Forest plots for sensitivity and specificity.

**Figure 4:**
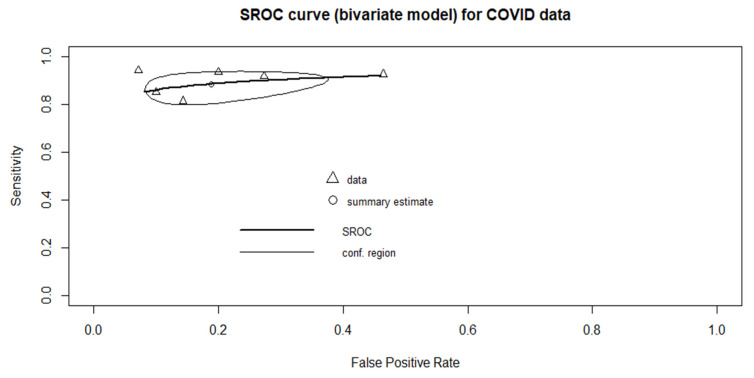
Summary receiver operating characteristic (sROC) curve, the AUC = 0.906.

Only six studies were included in the meta-analysis. The pooled sensitivity was 0.885 (95% CI: 0.818–0.929), and the pooled specificity was 0.811 (95% CI: 0.667–0.902). The heterogeneity of sensitivity was low (*p* = 0.434), whereas the heterogeneity of specificity was high (*p* = 0.000661). The pooled AUC was 0.906. The forest plots for sensitivity and specificity are provided in Figure 3. The sROC curve is shown in Figure 4.

### 3.4. Bias Assessment

**Figure 5:**
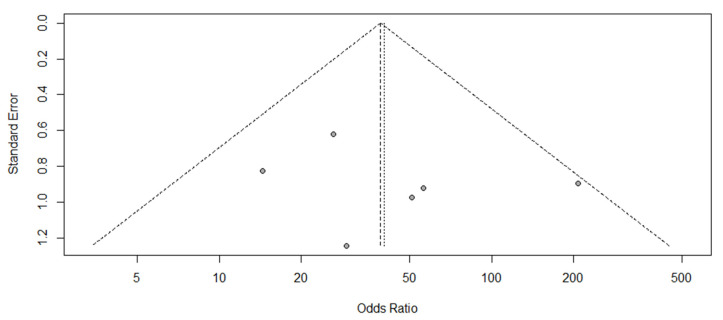
Funnel plot.

As shown in Figure 5, the publication bias was assessed using a funnel plot. The funnel plot result was symmetrical, indicating no obvious publication bias in this meta-analysis.

### 3.5. Review of Prediction Feature

**Figure 6:**
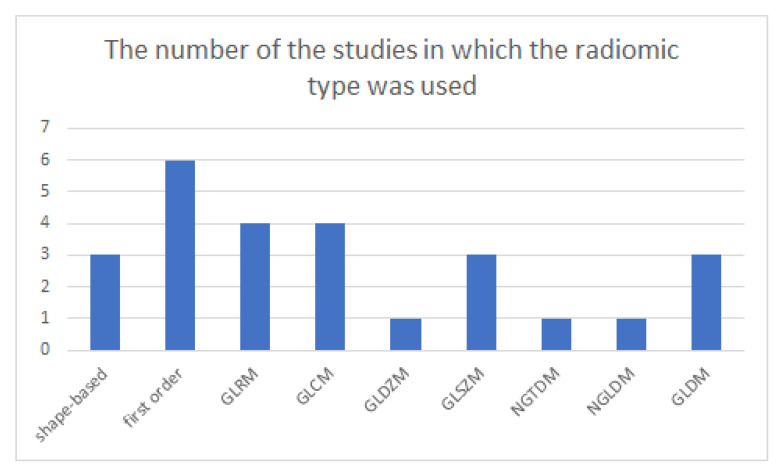
The number of the studies in which the radiomics type was used.

According to the International Symposium on Biomedical Imaging (ISBI), radiomics features can be classified as shape-based features, first-order features, gray-level run-length matrix (GLRM) features, gray-level co-occurrence matrix (GLCM) features, gray-level distance-zone matrix (GLDZM) features, gray-level size-zone matrix (GLSZM) features, neighborhood gray tone difference matrix (NGTDM) features, or neighboring gray-level dependence matrix (NGLDM) features [40]. Three studies used shape-based features, while all studies used first-order and second-order features. The details of used features are listed in Table 3. The number of the studies in which the radiomics type was used was shown in Figure 6.

### 3.6. Review of Prediction Models

Four studies used least absolute shrinkage and selection operator (LASSO) regression, one study used logistic regression, and one study used support vector machine (SVM) models with a radial basis function kernel. The details of the used models are listed in Table 4.

## 4. Discussion

This meta-analysis is the first to explore CT-based radiomics features for the differentiation of COVID-19 from other viral pneumonias. The advantage of conducting this meta-analysis is that a large number of patients were included. A total of 10,300 patients were included in this meta-analysis, increasing the robustness of our results. The prediction performance was fascinating with a pooled AUC of 0.906.

Among the studies that included viral pneumonia comparisons, two studies included only influenza infections [33,34]. Other studies included influenza and other viral pneumonia infections [35,36,37,38,39]. Considering that influenza also represents a highly contagious disease with a high prevalence of adult viral pneumonias [41,42]. We included the two studies which compared COVID-19 with influenza pneumonia in this meta-analysis [33,34].

The sensitivity of RT-PCR for COVID-19 diagnosis varied from 59 to 71% depending on viral load and test sample quality [2,5]. That is to say, an RT-PCR negative result was still shown in some COVID-19 infected patients [5,43]. Therefore, chest CT played a crucial role in the early diagnosis of COVID-19 pneumonia for the RT-PCR negative patients [44]. The identification of COVID-19 pneumonia on chest CT depended on radiologists’ interpretation. However, radiologists qualified only moderate sensitivity in differentiation COVID-19 from other viral pneumonia on chest CT [14]. With artificial intelligence assistance, radiologists achieved higher sensitivity in diagnosis of COVID-19 pneumonia [3,45]. In this meta-analysis comparing COVID-19 with other viral pneumonia under CT-based radiomics assistance, the pooled sensitivity was 0.885.

The radiomics quality scores in the included studies ranged from 13 to 16 points. The maximum RQS is 36 points. However, none of the collected studies were conducted prospectively, which led to a loss of 7 points. Thus, future studies should be conducted prospectively to achieve better-quality results.

Among the six studies included in the meta-analysis, four of them used LASSO regression. LASSO regression is a commonly used feature selection algorithm in the data science discipline. It is a logistic regression method with L1 regularization, which renders the prediction model more prone to noise, thus increasing its robustness [46]. One study used traditional logistic regression, whereas another used SVM. The SVM algorithm works well in the high-dimensional space, making it popular in machine learning.

First-order features, shape-based features, and second-order features were used in the prediction models. The power of radiomics features was displayed in many cancers [47,48,49]. However, this meta-analysis showed that radiomics features are useful not only in cancer, but also in other diseases, such as COVID-19 pneumonia.

The limitation of this meta-analysis is that all the studies were retrospective and conducted in China. However, there was no other suitable article that met the inclusion criteria in the three databases (Pubmed, Cochrane Library, and Embase) search process by two authors. One selected article was conducted in China but some of the involved patients were collected in the USA [39]. In the future, prospective and multinational studies should be conducted to validate the effectiveness of radiomics in COVID-19 detection using CT scans.

## 5. Conclusions

Our meta-analysis showed that CT-based radiomics feature models can successfully be used to differentiate COVID-19 from other viral pneumonias.

## Figures and Tables

**Table 1:** Details of the chosen studies.

Author Nation, Year	Study Type	ROI	Dataset	Training set	Internal Validation	External Validation	Highest AUC (95% CI)
Zheng [33] China, 2020	Retrospective observational	Pneumonia	COVID-19/IP	78	10-fold cross-validation	No	0.87 (0.77– 0.93)
Jin [34] China, 2020	Retrospective observational	Pneumonia	COVID-19/IP	2688	2688	2539 + 1110	0.9585 (0.9413– 0.9813) *
Fang [35] China, 2020	Retrospective cross-sectional	Pneumonia	COVID-19/VP	239	90	No	0.955 (0.899– 0.995)
Huang [36] China, 2020	Retrospective observational	Pneumonia	COVID-19/VP	126	55	No	0.956
Chen [37] China, 2020	Retrospective observational	Pneumonia	COVID-19/VP	114	23	No	0.968 (0.911–1.000)
Liu [38] China, 2021	Retrospective observational	Pneumonia	COVID-19/VP	379	131	40	0.93
Wang [39] China, 2020	Retrospective observational	Pneumonia	COVID-19/VP	9573 ^#^	1209 + 1219 ^#^	3799 ^#^	0.87

Note: COVID-19, coronavirus disease 2019; ROI, region of interest; AUC, area under the receiver operating characteristic curve; CI, confidence interval; IP, influenza pneumonia; VP, viral pneumonia.* The highest AUC in Jin’s study was based on a smaller cohort (*n* = 50) comprising only COVID-19 and influenza patients. ^#^ The number listed in Wang’s study is the CT scan slice number; thus, the results were not included in the meta-analysis.

**Table 2:** Radiomics quality scores of the included studies.

Study Criteria	Zheng [33] 2020	Jin [34] 2020	Fang [35] 2020	Huang [36] 2020	Chen [37] 2020	Liu [38] 2021	Wang [39] 2020
Image protocol quality	+1	+0	+1	+1	+1	+1	+1
Multiple segmentations	+1	+0	+0	+0	+1	+1	+1
Phantom study on all scanners	+0	+0	+0	+0	+0	+0	+0
Imaging at multiple time points	+0	+1	+0	+0	+0	+0	+0
Feature reduction or adjustment for multiple testing	+3	+3	+3	+3	+3	+3	+3
Multivariable analysis with non-radiomics features	+0	+0	+1	+1	+1	+1	+0
Detect and discuss biological correlates	+0	+0	+0	+0	+0	+0	+0
Cutoff analyses	+1	+1	+0	+1	+1	+0	+0
Discrimination statistics	+2	+1	+2	+2	+2	+2	+1
Calibration statistics	+1	+0	+2	+1	+0	+1	+0
Prospective study registered in a trial database	+0	+0	+0	+0	+0	+0	+0
Validation	+2	+4	+2	+2	+2	+2	+2
Comparison to “gold standard”	+0	+2	+2	+0	+0	+2	+0
Potential clinical utility	+2	+2	+2	+2	+2	+2	+2
Cost-effectiveness analysis	+0	+0	+0	+0	+0	+0	+0
Open science and data	+0	+1	+0	+0	+0	+0	+1
Total score (Maximum:36)	+13	+16	+15	+13	+14	+15	+13

**Table 3:** Features used in the prediction models.

Author Nation, Year	Radiomics Feature	Non-Radiomics Feature
Zheng [33] China, 2020	Shape-based, first-order, GLRM, GLDZM, NGLDM	Nil
Jin [34] China, 2020	First-order, GLCM, GLSZM, GLRM, NGTDM, GLDM *	Nil
Fang [35] China, 2020	First-order, GLCM	Lesion distribution, pleural effusion, maximum lesion range, mediastinal and hilar lymph node enlargement,
Huang [36] China, 2020	Shape-based, first-order, GLCM, GLDM *, GLSZM, GLRM	Halo sign, ground glass opacity (GGO), intralobular interstitial thickening (IIT)
Chen [37] China, 2020	Shape-based, first-order, GLSZM	Number of lesions with pleural thickening, white blood cell count, platelet count, number of lesions with crazy paving appearance
Liu [38] China 2021	first order, GLCM, GLDM*, GLRM	age, lesion distribution, neutrophil ratio, CT score, lymphocyte count

Note: GLRM, gray-level run-length matrix; GLCM, gray-level co-occurrence matrix; GLDZM, gray-level distance-zone matrix; GLSZM, gray-level size-zone matrix; NGTDM, neighborhood gray tone difference matrix; NGLDM, neighboring gray-level dependence matrix. * The gray-level dependence matrix (GLDM) is not listed by the International Symposium on Biomedical Imaging (ISBI).

**Table 4:** Prediction models used in the collected studies.

Author Nation, Year	Prediction Model
Zheng [33] China, 2020	LASSO regression
Jin [34] China, 2020	LASSO regression
Fang [35] China, 2020	LASSO regression
Huang [36] China, 2020	logistic regression
Chen [37] China, 2020	SVM models with a radial basis function kernel
Liu [38] China,2021	mRMR, LASSO regression

Note: LASSO, least absolute shrinkage and selection operator; SVM, support vector machine; mRMR: minimum redundancy and maximum relevance.

## Data Availability

All the data used were presented in the article.

## References

[1] Li Z., Yi Y., Luo X., Xiong N., Liu Y., Li S., Sun R., Wang Y., Hu B., Chen W. (2020). Development and clinical application of a rapid IgM-IgG combined antibody test for SARS-CoV-2 infection diagnosis. J. Med. Virol..

[2] Fang Y., Zhang H., Xie J., Lin M., Ying L., Pang P., Ji W. (2020). Sensitivity of Chest CT for COVID-19: Comparison to RT-PCR. Radiology.

[3] Bai H.X., Wang R., Xiong Z., Hsieh B., Chang K., Halsey K., Tran T.M.L., Choi J.W., Wang D.-C., Shi L.-B. (2020). Artificial Intelligence Augmentation of Radiologist Performance in Distinguishing COVID-19 from Pneumonia of Other Origin at Chest CT. Radiology.

[4] Liu R., Han H., Liu F., Lv Z., Wu K., Liu Y., Feng Y., Zhu C. (2020). Positive rate of RT-PCR detection of SARS-CoV-2 infection in 4880 cases from one hospital in wuhan, china, from jan to feb 2020. Clin. Chim. Acta.

[5] Ai T., Yang Z., Hou H., Zhan C., Chen C., Lv W., Tao Q., Sun Z., Xia L. (2020). Correlation of Chest CT and RT-PCR Testing for Coronavirus Disease 2019 (COVID-19) in China: A Report of 1014 Cases. Radiology.

[6] Chung M., Bernheim A., Mei X., Zhang N., Huang M., Zeng X., Cui J., Xu W., Yang Y., Fayad Z.A. (2020). CT Imaging Features of 2019 Novel Coronavirus (2019-nCoV). Radiology.

[7] Stephanie S., Shum T., Cleveland H., Challa S.R., Herring A., Jacobson F.L., Hatabu H., Byrne S.C., Shashi K., Araki T. (2020). Determinants of chest x-ray sensitivity for covid-19: A multi-institutional study in the united states. Radiol. Cardiothorac. Imaging.

[8] Pan Y., Guan H., Zhou S., Wang Y., Li Q., Zhu T., Hu Q., Xia L. (2020). Initial CT findings and temporal changes in patients with the novel coronavirus pneumonia (2019-nCoV): A study of 63 patients in Wuhan, China. Eur. Radiol..

[9] Rong X., Yang L., Chu H., Fan M. (2020). Effect of delay in diagnosis on transmission of COVID-19. Math. Biosci. Eng..

[10] Hussain L., Nguyen T., Li H., Abbasi A.A., Lone K.J., Zhao Z., Zaib M., Chen A., Duong T.Q. (2020). Machine-learning classification of texture features of portable chest X-ray accurately classifies COVID-19 lung infection. Biomed. Eng. Online.

[11] Khuzani A.Z., Heidari M., Shariati S.A. (2021). COVID-Classifier: An automated machine learning model to assist in the diagnosis of COVID-19 infection in chest X-ray images. Sci. Rep..

[12] Kikkisetti S., Zhu J., Shen B., Li H., Duong T.Q. (2020). Deep-learning convolutional neural networks with transfer learning accurately classify COVID-19 lung infection on portable chest radiographs. PeerJ.

[13] López-Cabrera J.D., Orozco-Morales R., Portal-Diaz J.A., Lovelle-Enríquez O., Pérez-Díaz M. (2021). Current limitations to identify COVID-19 using artificial intelligence with chest X-ray imaging. Health Technol..

[14] Bai H.X., Hsieh B., Xiong Z., Halsey K., Choi J.W., Tran T.M.L., Pan I., Shi L.-B., Wang D.-C., Mei J. (2020). Performance of Radiologists in Differentiating COVID-19 from Non-COVID-19 Viral Pneumonia at Chest CT. Radiology.

[15] Yip S.S.F., Aerts H.J.W.L. (2016). Applications and limitations of radiomics. Phys. Med. Biol..

[16] Liu Z., Wang S., Dong D., Wei J., Fang C., Zhou X., Sun K., Li L., Li B., Wang M. (2019). The Applications of Radiomics in Precision Diagnosis and Treatment of Oncology: Opportunities and Challenges. Theranostics.

[17] Tan H.-B., Xiong F., Jiang Y.-L., Huang W.-C., Wang Y., Li H.-H., You T., Fu T.-T., Lu R., Peng B.-W. (2020). The study of automatic machine learning base on radiomics of non-focus area in the first chest CT of different clinical types of COVID-19 pneumonia. Sci. Rep..

[18] Chen H., Zeng M., Wang X., Su L., Xia Y., Yang Q., Liu D. (2021). A CT-based radiomics nomogram for predicting prognosis of coronavirus disease 2019 (COVID-19) radiomics nomogram predicting COVID-19. Br. J. Radiol..

[19] Yue H., Yu Q., Liu C., Huang Y., Jiang Z., Shao C., Zhang H., Ma B., Wang Y., Xie G. (2020). Machine learning-based CT radiomics method for predicting hospital stay in patients with pneumonia associated with SARS-CoV-2 infection: A multicenter study. Ann. Transl. Med..

[20] Liu C., Wang X., Liu C., Sun Q., Peng W. (2020). Differentiating novel coronavirus pneumonia from general pneumonia based on machine learning. Biomed. Eng. Online.

[21] Li L., Qin L., Xu Z., Yin Y., Wang X., Kong B., Bai J., Lu Y., Fang Z., Song Q. (2020). Using Artificial Intelligence to Detect COVID-19 and Community-acquired Pneumonia Based on Pulmonary CT: Evaluation of the Diagnostic Accuracy. Radiology.

[22] Fowlkes A., Steffens A., Temte J., Di Lonardo S., McHugh L., Martin K., Rubino H., Feist M., Davis C., Selzer C. (2015). Incidence of medically attended influenza during pandemic and post-pandemic seasons through the Influenza Incidence Surveillance Project, 2009–2013. Lancet Respir. Med..

[23] Moriyama M., Hugentobler W.J., Iwasaki A. (2020). Seasonality of Respiratory Viral Infections. Annu. Rev. Virol..

[24] Li Y., Reeves R.M., Wang X., Bassat Q., Brooks W.A., Cohen C., Moore D.P., Nunes M., Rath B., Campbell H. (2019). Global patterns in monthly activity of influenza virus, respiratory syncytial virus, parainfluenza virus, and metapneumovirus: A systematic analysis. Lancet Glob. Health.

[25] Zhao W., Zhong Z., Xie X., Yu Q., Liu J. (2020). Relation Between Chest CT Findings and Clinical Conditions of Coronavirus Disease (COVID-19) Pneumonia: A Multicenter Study. Am. J. Roentgenol..

[26] Gillies R.J., Kinahan P.E., Hricak H. (2016). Radiomics: Images Are More than Pictures, They Are Data. Radiology.

[27] Xie C., Ng M.-Y., Ding J., Leung S.T., Lo C.S.Y., Wong H.Y.F., Vardhanabhuti V. (2020). Discrimination of pulmonary ground-glass opacity changes in COVID-19 and non-COVID-19 patients using CT radiomics analysis. Eur. J. Radiol. Open.

[28] McInnes M.D.F., Moher D., Thombs B.D., McGrath T.A., Bossuyt P.M., Clifford T., Cohen J.F., Deeks J.J., Gatsonis C., Hooft L. (2018). Preferred reporting items for a systematic review and meta-analysis of diagnostic test accuracy studies: The PRISMA-DTA statement. JAMA.

[29] Zwanenburg A., Vallières M., Abdalah M.A., Aerts H.J.W.L., Andrearczyk V., Apte A., Ashrafinia S., Bakas S., Beukinga R.J., Boellaard R. (2020). The Image Biomarker Standardization Initiative: Standardized Quantitative Radiomics for High-Throughput Image-based Phenotyping. Radiology.

[30] Song J., Wang H., Liu Y., Wu W., Dai G., Wu Z., Zhu P., Zhang W., Yeom K.W., Deng K. (2020). End-to-end automatic differentiation of the coronavirus disease 2019 (COVID-19) from viral pneumonia based on chest CT. Eur. J. Nucl. Med. Mol. Imaging.

[31] Huang Y., Zhang Z., Liu S., Li X., Yang Y., Ma J., Li Z., Zhou J., Jiang Y., He B. (2021). CT-based radiomics combined with signs: A valuable tool to help radiologist discriminate COVID-19 and influenza pneumonia. BMC Med. Imaging.

[32] Wang L., Kelly B., Lee E.H., Wang H., Zheng J., Zhang W., Halabi S., Liu J., Tian Y., Han B. (2021). Multi-classifier-based identification of COVID-19 from chest computed tomography using generalizable and interpretable radiomics features. Eur. J. Radiol..

[33] Zeng Q.Q., Zheng K.I., Chen J., Jiang Z.H., Tian T., Wang X.B., Ma H.L., Pan K.H., Yang Y.J., Chen Y.P. (2020). Radiomics-based model for accurately distinguishing between severe acute respiratory syndrome associated coronavirus 2 (SARS-CoV-2) and influenza A infected pneumonia. MedComm.

[34] Jin C., Chen W., Cao Y., Xu Z., Tan Z., Zhang X., Deng L., Zheng C., Zhou J., Shi H. (2020). Development and evaluation of an artificial intelligence system for COVID-19 diagnosis. Nat. Commun..

[35] Fang X., Li X., Bian Y., Ji X., Lu J. (2020). Radiomics nomogram for the prediction of 2019 novel coronavirus pneumonia caused by SARS-CoV-2. Eur Radiol..

[36] Huang Y., Zhang Z., Li X., Yang Y., Li Z., Zhou J., Jiang Y., Ma J., Liu S., Bo H. (2020). CT-based radiomics combined with signs: A valuable tool to help physician discriminate COVID-19 and other viral pneumonia. Eur. PMC.

[37] Chen H.J., Chen Y., Yuan L., Wang F., Mao L., Li X., Cai Q., Qiu J., Tian J., Chen F. (2020). Machine learning-based CT radiomics model distinguishes COVID-19 from other viral pneumonia. Res. Sq..

[38] Liu H., Ren H., Wu Z., Xu H., Zhang S., Li J., Hou L., Chi R., Zheng H., Chen Y. (2021). CT radiomics facilitates more accurate diagnosis of COVID-19 pneumonia: Compared with CO-RADS. J. Transl. Med..

[39] Wang H., Wang L., Lee E.H., Zheng J., Zhang W., Halabi S., Liu C., Deng K., Song J., Yeom K.W. (2020). Decoding COVID-19 pneumonia: Comparison of deep learning and radiomics CT image signatures. Eur. J. Nucl. Med. Mol. Imaging.

[40] Tibshirani R. (1996). Regression Shrinkage and Selection Via the Lasso. J. R. Stat. Soc. Ser. B Statistical Methodol..

[41] Jain S. (2017). Epidemiology of Viral Pneumonia. Clin. Chest Med..

[42] Burk M., El-Kersh K., Saad M., Wiemken T., Ramirez J., Cavallazzi R. (2016). Viral infection in community-acquired pneumonia: A systematic review and meta-analysis. Eur. Respir. Rev..

[43] Korkmaz I., Dikmen N., Keleş F.O., Bal T. (2021). Chest CT in COVID-19 pneumonia: Correlations of imaging findings in clinically suspected but repeatedly RT-PCR test-negative patients. Egypt. J. Radiol. Nucl. Med..

[44] Chen H.J., Qiu J., Wu B., Huang T., Gao Y., Wang Z.P., Chen Y., Chen F. (2020). Early chest CT features of patients with 2019 novel coronavirus (COVID-19) pneumonia: Relationship to diagnosis and prognosis. Eur. Radiol..

[45] Yousefzadeh M., Esfahanian P., Movahed S.M.S., Gorgin S., Rahmati D., Abedini A., Nadji S.A., Haseli S., Karam M.B., Kiani A. (2021). ai-corona: Radiologist-assistant deep learning framework for COVID-19 diagnosis in chest CT scans. PLoS ONE.

[46] Lambin P., Leijenaar R.T., Deist T.M., Peerlings J., De Jong E.E., Van Timmeren J., Sanduleanu S., LaRue R.T., Even A.J., Jochems A. (2017). Radiomics: The bridge between medical imaging and personalized medicine. Nat. Rev. Clin. Oncol..

[47] Ursprung S., Beer L., Bruining A., Woitek R., Stewart G.D., Gallagher F., Sala E. (2020). Radiomics of computed tomography and magnetic resonance imaging in renal cell carcinoma—a systematic review and meta-analysis. Eur. Radiol..

[48] Kothari G., Korte J., Lehrer E.J., Zaorsky N.G., Lazarakis S., Kron T., Hardcastle N., Siva S. (2021). A systematic review and meta-analysis of the prognostic value of radiomics based models in non-small cell lung cancer treated with curative radiotherapy. Radiother. Oncol..

[49] Kao Y.-S., Hsu Y. (2021). A Meta-Analysis for Using Radiomics to Predict Complete Pathological Response in Esophageal Cancer Patients Receiving Neoadjuvant Chemoradiation. In Vivo.

